# A Common Complement C3 Variant Is Associated with Protection against Wet Age-Related Macular Degeneration in a Japanese Population

**DOI:** 10.1371/journal.pone.0028847

**Published:** 2011-12-12

**Authors:** Suiho Yanagisawa, Naoshi Kondo, Akiko Miki, Wataru Matsumiya, Sentaro Kusuhara, Yasutomo Tsukahara, Shigeru Honda, Akira Negi

**Affiliations:** Department of Surgery, Division of Ophthalmology, Kobe University Graduate School of Medicine, Kobe, Japan; Medical College of Wisconsin, United States of America

## Abstract

**Background:**

Genetic variants in the complement component 3 gene (*C3*) have been shown to be associated with age-related macular degeneration (AMD) in Caucasian populations of European descent. In particular, a nonsynonymous coding variant, rs2230199 (R102G), is presumed to be the most likely causal variant in the *C3* locus based on strong statistical evidence for disease association and mechanistic functional evidence. However, the risk allele is absent or rare (<1%) in Japanese and Chinese populations, and the association of R102G with AMD has not been reported in Asian populations. Genetic heterogeneity of disease-associated variants among different ethnicities is common in complex diseases. Here, we sought to examine whether other common variants in *C3* are associated with wet AMD, a common advanced-stage manifestation of AMD, in a Japanese population.

**Methodology/Principal Findings:**

We genotyped 13 tag single nucleotide polymorphisms (SNPs) that capture the majority of common variations in the *C3* locus and tested for associations between these SNPs and wet AMD in a Japanese population comprising 420 case subjects and 197 controls. A noncoding variant in *C3* (rs2241394) exhibited statistically significant evidence of association (allelic *P* = 8.32×10^−4^; odds ratio = 0.48 [95% CI = 0.31–0.74] for the rs2241394 C allele). Multilocus logistic regression analysis confirmed that the effect of rs2241394 was independent of the previously described loci at *ARMS2* and *CFH*, and that the model including variants in *ARMS2* and *CFH* plus *C3* rs2241394 provided a better fit than the model without rs2241394. We found no evidence of epistasis between variants in *C3* and *CFH*, despite the fact that they are involved in the same biological pathway.

**Conclusions:**

Our study provides evidence that *C3* is a common AMD-associated locus that transcends racial boundaries and provides an impetus for more detailed genetic characterization of the *C3* locus in Asian populations.

## Introduction

Age-related macular degeneration (AMD) is a common multifactorial and heterogeneous disorder, characterized by progressive degeneration of the central region of the retina (macula) [1], [2]. Pigmentary abnormalities of the retinal pigment epithelium (RPE) and extracellular deposits (drusen) under the retina are among the early-stage manifestations of AMD. As the condition progresses, extensive atrophy of the RPE and outer retina (geographic atrophy or dry AMD) or abnormal vessel growth underneath the macula (exudative or wet AMD) are common advanced-stage manifestations. AMD affects 30–50 million individuals worldwide and is a leading cause of legal blindness among older individuals in developed countries [1], [2].

Although the precise etiology of AMD remains elusive, genetic studies have provided significant insights into the molecular basis of AMD. Several genes encoding proteins involved in the complement pathway have been shown to be associated with susceptibility to AMD, including the complement factor H gene (*CFH*) on chromosome 1q32 [3]–[5], two neighboring genes, complement component 2 (*C2*) and complement factor B (*CFB*) on 6p21 [6]–[8], the complement factor I gene (*CFI*) on 4q25 [9], [10], and the complement component 3 gene (*C3*) on 19p13 [11]–[13]. These findings strongly implicate aberrant regulation and/or activation of the complement pathway in the mechanism of susceptibility to AMD. In addition to the association with complement pathway genes, AMD has been convincingly shown to be associated with two adjacent genes on 10q26 (age-related maculopathy susceptibility 2 [*ARMS2*] and high-temperature requirement factor H [*HTRA1*]) [14]–[16], which together account for nearly half of the heritability of AMD [7].

AMD susceptibility loci have been primarily discovered in populations of European descent, of which only the association of *CFH* [17]–[20] and the *ARMS2*/*HTRA1* loci [14], [21], [22] have been convincingly validated in Asian populations. We recently reported a significant association of wet AMD in a Japanese population with the same susceptibility variant near *CFI* as that observed in individuals of European descent [23], indicating that, along with *CFH* and *ARMS2*/*HTRA1*, *CFI* is a susceptibility locus of AMD that transcends racial boundaries. However, studies have also revealed the existence of genetic heterogeneity in AMD susceptibility at the *C3* locus between populations of European and Asian descent. A nonsynonymous coding variant in *C3*, rs2230199 (R102G), was consistently found to be associated with AMD in Caucasian populations [11]–[13], [24], [25], but not in Asians [25]–[28]. Furthermore, the allelic frequency of the R102G variant is absent in Japanese and rare (<1%) in Chinese populations, according to the data from the International HapMap Project and published studies [25], [27], [28], [29], while risk allele frequency is almost 20% in individuals of European descent [25]. It has been proposed that genetic effects of disease-associated variants are similar across racial boundaries regardless of their widely divergent allelic frequency between different populations [30]. However, it has also been documented that genetic heterogeneity of disease susceptibility between ethnic groups is common in complex diseases [31], [32], and thus, disease-associated variants present in populations of European descent might not be applicable to Asian populations because of underlying genetic heterogeneity. Indeed, two recent studies have suggested a role for common intronic variants of the *C3* locus in susceptibility to wet AMD in Japanese and Chinese populations [26], [27], implying that more common *C3* variants are associated with the disease in Asians. Here we genotyped 13 tag single nucleotide polymorphisms (SNPs) that capture the majority of common variations in the *C3* locus and tested for associations between these SNPs and wet AMD in a Japanese population comprising 420 case subjects and 197 controls.

## Materials and Methods

### Ethics Statement

The study protocol was approved by the Institutional Review Board at Kobe University Graduate School of Medicine and performed in accordance with the Declaration of Helsinki. Written informed consent was obtained from all subjects before participation in this study.

### Study participants

All cases and controls included in this study were Japanese individuals recruited from the Department of Ophthalmology at Kobe University Hospital in Kobe, Japan. The demographic details of the study population are shown in Table 1. All cases and control subjects underwent comprehensive ophthalmic examination, including visual acuity measurement, slit-lamp examination, and dilated funduscopic examination. Fundus findings in each eye were classified according to the clinical age-related maculopathy staging system (CARMS) [33] as previously described [7], [12]. All of our case subjects had wet AMD and associated manifestations such as nondrusenoid pigment epithelial detachment, serous or hemorrhagic retinal detachment, and subretinal or sub-RPE hemorrhages and fibrosis; they were categorized as having CARMS stage 5 [33]. The controls were individuals aged 56 years or older and were defined as cases without macular degeneration and changes, such as drusen or pigment abnormalities. Thus, controls were categorized as having CARMS stage 1 [33] on the basis of comprehensive ophthalmic examinations.

**Table 1:** Characteristics of the study population.

	Wet AMD	Controls
Number of subjects	420	197
Gender (male/female)	331/89	117/80
Mean age ± SD (years)	74±7.5	72±6.0
Age range (years)	54–94	56–95

AMD: age-related macular degeneration; SD: standard deviation.

### Genotyping

Genomic DNA was extracted from peripheral blood using standard methodology. Genotyping was performed using the TaqMan® SNP Genotyping Assays (Applied Biosystems, Foster City, CA) on a StepOnePlus™ Real-Time PCR System (Applied Biosystems) in accordance with the manufacturer's recommendations.

### SNP selection

To comprehensively yet efficiently screen *C3* sequences for common genetic variations, tag SNPs were selected from the HapMap Project database for the Japanese in Tokyo (JPT) population using the tag selection tool. Thirteen tag SNPs were selected for genotyping, which captured 29 of 34 SNPs in the *C3* locus exhibiting a minor frequency greater than 10% with a mean r^2^ value of 0.986.

### Statistical analysis

Allelic associations were evaluated for each SNP by chi-square tests on 2×2 contingency tables using the software package PLINK v1.00 (http://pngu.mgh.harvard.edu/purcell/plink/) [34]. The odds ratio (OR) and corresponding 95% confidence interval (CI) were calculated relative to the major allele. In addition to obtaining nominal *P* values, corrected empirical *P* values for multiple testing were generated by 10,000 permutation tests using the Max (T) permutation procedure implemented in PLINK [34]. We also applied a Bonferroni correction, where nominal *P*-values were multiplied by 13 (the number of SNPs tested for association). To adjust for age and gender differences between the case and control subjects, logistic regression analysis was performed using SNPStats (http://bioinfo.iconcologia.net/SNPStats), with age and gender controlled as covariates. Age and gender were included in this model as a continuous covariate measured in years and a categorical covariate, respectively. Deviations from the Hardy–Weinberg equilibrium were tested using the exact test implemented in PLINK [35]. Haploview software was used to assess linkage disequilibrium (LD) patterns and haplotype association statistics [36]. Haplotype blocks were determined using the solid spine of LD algorithm with a minimum D' of 0.8. To correct for multiple testing in the haplotype association analysis, 10,000 permutations were run using this software. An omnibus (or global) test of the haplotype association was performed with PLINK. To determine whether a single variant could explain an entire omnibus haplotype association, conditional haplotype-based likelihood ratio tests implemented in PLINK were conducted. The haplotype association was assessed further using sliding window analyses of four adjacent SNPs across the *C3* region. For this analysis, sliding windows of overlapping haplotypes were tested in sequence. For example, SNPs rs2250656, rs2230205, rs11569429, and rs11672613 were treated as a single haplotype, followed by SNPs rs2230205, rs11569429, rs11672613, and rs428453. The significance values were evaluated on the basis of omnibus test *P* values. The sliding window analyses were conducted using the PLINK software. The FASTSNP program (http://fastsnp.ibms.sinica.edu.tw/pages/input_CandidateGeneSearch.jsp) was used to predict the function of a SNP of interest [37].

To examine a genetic effect detected here in the context of three validated AMD-risk loci for Asians (the A69S variant [rs10490924] in *ARMS2* [14], [21], [22] and the I62V variant [rs800292] and Y402H variant [rs1061170] in *CFH* [17]–[20]), we conducted logistic regression analyses with the R statistical analysis package (http://www.r-project.org/). For each locus, the genetic model of best fit was determined before genotypes were coded according to additive, dominant, and recessive models. Akaike Information Criterion (AIC) was used to select the model of best fit. The best models for each locus were then combined into multilocus models, and an effect of the *C3* variant after controlling for *ARMS2* A69S, *CFH* I62V, and *CFH* Y402H was estimated. Furthermore, we compared two logistic regression models (the full model including all four variants versus a reduced model in which the C3 variant was omitted) by using a likelihood ratio test and calculating AIC values. To determine epistatic effects between *C3* rs2241394 and *CFH* I62V or Y402H, pairwise interaction analysis was performed using the epistasis option in PLINK.

## Results

None of the 13 SNPs reported in the present study showed significant deviation from the Hardy–Weinberg equilibrium in both the case and control subjects (*P*>0.05). Marker information, allelic frequencies, and summary statistics for all evaluated SNPs are shown in Table 2. In single-SNP analyses, two of the 13 SNPs showed nominally significant associations with wet AMD (rs2241394, nominal *P* = 8.32×10^−4^; rs428453, nominal *P* = 0.0240), of which only rs2241394 withstood multiple test corrections (corrected empirical *P* = 0.0102; Bonferroni-corrected *P* = 0.0108, Table 2). The minor allele C of rs2241394 was associated with protection against the disease, with a frequency of 0.052 in disease cases and 0.104 in controls (per allele OR = 0.48 [95% CI = 0.31–0.74]; Table 2). In a dominant genetic model, OR for individuals carrying at least one copy of the protective allele was 0.45 (95% CI = 0.28–0.72; *P* = 7.81×10^−4^). Inclusion of age and gender as covariates in the logistic regression model did not substantially change the significance of the association (age- and gender-adjusted OR = 0.48 [95% CI = 0.30–0.75], *P* = 0.0016, additive model; age- and gender-adjusted OR = 0.44 [95% CI = 0.27–0.72], *P* = 0.0012, dominant model).

**Table 2:** Results of single-marker association test.

			Minor allele frequency		Association Results		
SNP (location)	Position in NCBI build 36.3	Minor allele	Cases	Controls	Allelic *P*-value	Allelic OR (95% CI)	Corrected empirical *P*-value*
rs2250656 (intron 2)	6658534 bp	C	0.230	0.241	0.660	0.94 (0.71–1.24)	1
rs2230205 (exon 14; T612T)	6649704 bp	T	0.413	0.406	0.816	1.03 (0.81–1.31)	1
rs11569429 (intron 14)	6649074 bp	T	0.132	0.150	0.403	0.86 (0.61–1.22)	0.995
rs11672613 (intron 17)	6645246 bp	C	0.470	0.452	0.544	1.08 (0.85–1.37)	1
rs428453 (exon 19; V807V)	6642157 bp	C	0.096	0.140	0.0240	0.66 (0.46–0.95)	0.225
rs432001 (intron 24)	6633683 bp	G	0.152	0.155	0.912	0.98 (0.70–1.37)	1
rs7257062 (intron 29)	6625945 bp	C	0.241	0.211	0.247	1.19 (0.89–1.58)	0.929
rs2241393 (intron 29)	6625304 bp	G	0.329	0.305	0.40	1.12 (0.86–1.45)	0.995
rs2241394 (intron 29)	6625230 bp	C	0.052	0.104	8.32×10^−4^	0.48 (0.31–0.74)	0.0102
rs1389623 (intron 33)	6624197 bp	A	0.082	0.102	0.263	0.79 (0.53–1.19)	0.942
rs7951 (exon 35; A1437A)	6621991 bp	A	0.082	0.102	0.263	0.79 (0.53–1.19)	0.942
rs344555 (intron 37)	6619360 bp	T	0.385	0.343	0.156	1.20 (0.93–1.54)	0.802
rs11569562 (intron 38)	6618753 bp	G	0.477	0.515	0.215	0.86 (0.68–1.09)	0.897

OR: odds ratio; CI: confidence intervals.

*Empirical *P*-values corrected for multiple testing (corrected empirical *P*-values) were generated by 10,000 permutation tests using Max (T) permutation procedure implemented in the PLINK software.

**Figure 1:**
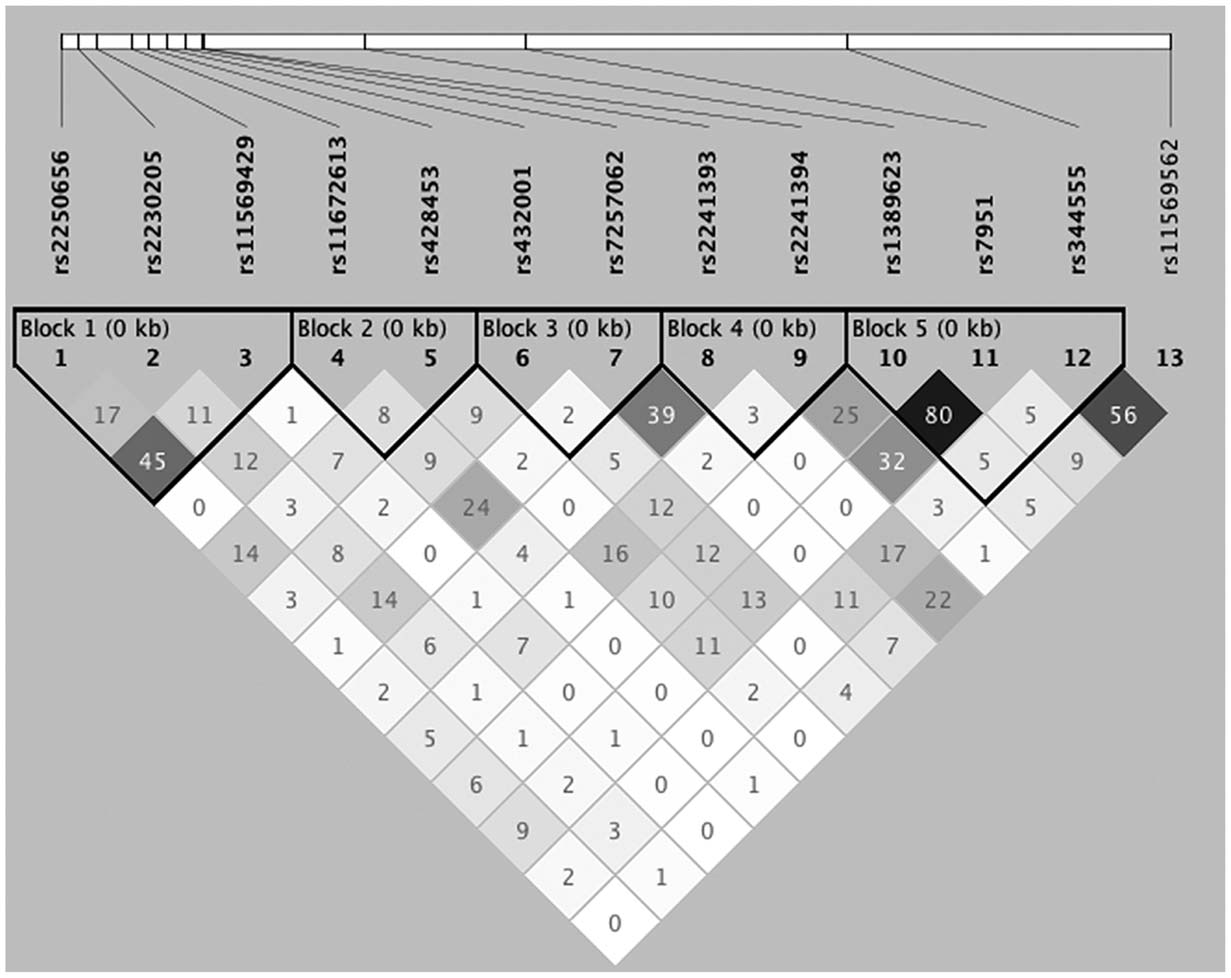
Linkage disequilibrium (LD) structure of the *C3* locus. LD was measured using data from all subjects in the present study. The haplotype blocks were determined by the solid spine of LD method implemented in the Haploview software. Each box provides estimated statistics of the coefficient of determination (r^2^), with darker shades representing stronger LD.

The pairwise LD structure was constructed with the 13 SNPs genotyped (Figure 1). Five haplotype blocks were defined, and association with the disease was restricted to block 4 where the disease-associated SNP rs2241394 resided as demonstrated by the significant omnibus result (omnibus *P* = 0.00367 at 2 degrees of freedom, Table 3). Only one haplotype in block 4 was found to be significantly associated with the disease, with a haplotype frequency of 0.052 in affected individuals and 0.104 in controls (*P* = 8.0×10^−4^; OR = 0.48 [95% CI = 0.31–0.74]; Table 3). This association remained statistically significant after correction for multiple testing (permutation *P* = 0.011). The disease-associated haplotype was completely described by the protective allele C of rs2241394, and a conditional haplotype-based likelihood ratio test revealed that the significant omnibus haplotype association detected in haplotype block 4 disappeared when it was estimated to be conditional on rs2241394 (omnibus *P* = 0.85), confirming that rs2241394 is responsible for the haplotype association detected in this LD block. To further assess haplotype associations, we conducted a sliding window analysis of four adjacent SNPs across the *C3* region. Significant associations were observed only around rs2241394 (Table 4), and the strongest association was found when four variants—rs2241393, rs2241394, rs1389623, and rs7951—were included together (omnibus *P* = 9.81×10^−4^, Table 4).

**Table 3:** Association of *C3* haplotype blocks with wet AMD.

		Frequency				
	Haplotype	Cases	Controls	*P*-value*	OR (95% CI)	Omnibus *P*-value†
Block 1rs2250656|rs2230205|rs11569429	TTC	0.401	0.399	0.952	1.01 (0.79–1.29)	0.857
	TCC	0.362	0.351	0.699	1.05 (0.82–1.35)	
	CCT	0.125	0.141	0.442	0.86 (0.61–1.22)	
	CCC	0.092	0.093	0.967	0.99 (0.65–1.49)	
	CTC	0.012	0.0007	0.399	1.57 (0.43–5.74)	
Block 2rs11672613|rs428453	CG	0.468	0.442	0.388	1.11 (0.87–1.41)	0.153
	TG	0.435	0.419	0.576	1.07 (0.84–1.37)	
	TC	0.094	0.130	0.0595	0.70 (0.48–1.02)	
Block 3rs432001|rs7257062	AT	0.619	0.643	0.420	0.91 (0.71–1.16)	0.691
	AC	0.229	0.202	0.299	1.16 (0.87–1.56)	
	GT	0.140	0.146	0.779	0.95 (0.67–1.33)	
	GC	0.012	0.008	0.574	1.57 (0.43–5.74)	
Block 4rs2241393|rs2241394	CG	0.619	0.591	0.353	1.12 (0.88–1.43)	0.00367
	GG	0.329	0.305	0.40	1.12 (0.86–1.45)	
	CC	0.052	0.104	8.0×10^−4^	0.48 (0.31–0.74)	
Block 5rs1389623|rs7951|rs344555	GGC	0.521	0.556	0.260	0.87 (0.68–1.11)	0.0846
	GGT	0.385	0.343	0.156	1.20 (0.93–1.54)	
	AAC	0.070	0.101	0.0596	0.67 (0.44–1.02)	

OR: odds ratio; CI: confidence intervals.

The association of haplotype CC in block 4 remained statistically significant after correction for multiple testing (permutation *P* = 0.011).

*The *P*-values were calculated by the chi-square test on haplotype counts (1 degree of freedom).

† The omnibus *P*-values were calculated by the PLINK software (4 degrees of freedom for block 1; 2 degrees of freedom for block 2, 4, and 5; 3 degrees of freedom for block 3).

**Table 4:** Four-marker sliding window haplotype analysis over the entire *C3* locus.

	Omnibus *P* Value*
SNP	4-Marker
rs2250656	0.959
rs2230205	0.706
rs11569429	0.476
rs11672613	0.503
rs428453	0.269
rs432001	0.0515
rs7257062	0.0555
rs2241393	9.81×10^−4^
rs2241394	0.00103
rs1389623	0.0738
rs7951	-
rs344555	-
rs11569562	-

SNP: single nucleotide polymorphism.

*Omnibus *P* value corresponding to the haplotype with the listed SNP as the first SNP in the haplotype.

To examine the possibility that the disease-associated SNP rs2241394 might be correlated with untyped SNPs, we investigated the LD structure across the genomic region extending approximately 200 kb upstream and downstream of the *C3* locus. Genotype data were retrieved from the 1000 Genome Project (August 2010 release) [38] and International HapMap (release 24) JPT+CHB datasets [39], and correlations (as defined by r^2^ values) were examined. In this genomic region, we found 594 SNPs but did not identify any SNPs that are highly correlated with rs2241394 (all pairwise r^2^<0.45).

Next, we examined the genetic effect of rs2241394 in the context of three validated AMD-risk loci for Asians (*ARMS2* A69S [14], [21], [22], *CFH* I62V [17]–[19], and *CFH* Y402H [20]). Using unconditional logistic regression, the genetic model of best fit for *C3* rs2241394, *ARMS2* A69S, *CFH* I62V, and *CFH* Y402H was determined and genotypes were coded according to additive, dominant, and recessive models. On the basis of AIC values, *ARMS2* A69S, and *CFH* I62V had the best fit under an additive model, and *C3* rs2241394 and *CFH* Y402H had the best fit under a dominant model. The best models were then combined into multilocus logistic regression models. After including the effects of *CFH* I62V, *CFH* Y402H, and *ARMS2* A69S, *C3* rs2241394 retained significant association (model 1; Table 5). Furthermore, we found that the model including all four variants–*C3* rs2241394, *ARMS2* A69S, *CFH* I62V, and *CFH* Y402H–fit significantly better than the model without *C3* rs2241394 (likelihood ratio test χ^2^ = 10.32, *P* = 0.00132, model 1 vs. model 2; AIC = 692.0 and 700.3 for model 1 and 2, respectively; Table 5).

**Table 5:** Multilocus logistic regression analysis of *C3* rs2241394, *ARMS2* A69S, *CFH* I62V, and *CFH* Y402H.

Model	Effect	*P*-value	OR (95% CI)	AIC
1	*C3* rs2241394	0.00125	0.43 (0.26–0.72)	692.0
	*ARMS2* A69S	2.30×10^−9^	2.15 (1.67–2.77)	
	*CFH* I62V	3.24×10^−5^	1.76 (1.35–2.30)	
	*CFH* Y402H	0.00302	2.34 (1.33–4.10)	
2	*ARMS2* A69S	1.66×10^−9^	2.15 (1.68–2.77)	700.3
	*CFH* I62V	1.87×10^−5^	1.78 (1.37–2.32)	
	*CFH* Y402H	0.00523	2.20 (1.26–3.82)	

AIC: Akaike information criterion.

Finally, we conducted pairwise interaction analysis to evaluate potential epistatic effects between *C3* rs2241394 and *CFH* I62V or *CFH* Y402H, because the proteins encoded by these loci biologically interact in the complement pathway [40]. However, we did not find any evidence of epistasis between rs2241394 and *CFH* variants (all *P*>0.05).

## Discussion

We genotyped 13 tag SNPs that capture the majority of common genetic variations in the *C3* locus and found statistically significant evidence for association between an intronic *C3* variant (rs2241394) and wet AMD in a Japanese population (*P* = 8.32×10^−4^). Haplotype analyses identified the LD block where rs2241394 resides as being the only significant locus, and haplotype association was completely explained by rs2241394. Logistic regression analysis showed that the effect of rs2241394 is independent of the established associations of *ARMS2* A69S, *CFH* I62V, and *CFH* Y402H, and that the model including these three established loci plus *C3* rs2241394 provides a better fit than the model without rs2241394. Although the proteins encoded by *C3* and *CFH* are involved in the same biological pathway, we found no evidence of epistasis between rs2241394 and the two *CFH* variants.

Complement has emerged as an important element in AMD pathology [41], [42], because of the identification of various complement-related molecules in drusen and nearby RPE [42]. In addition, recent successes in the identification of genetic susceptibility loci for AMD have revealed several molecules involved in the complement pathway, including CFH [3]–[5], CFB [6]–[8], C2 [6]–[8], CFI [9], [10], [23], and C3 [11]–[13]. Furthermore, systemic complement activation was observed in AMD patients [43]–[45] and nutritional supplementation with zinc was shown to delay the progression of AMD [46], an effect likely mediated by an inhibitory effect of zinc on complement activity [47]. C3 is a central component of all three pathways of complement activation: the alternative, classical, and mannose-binding lectin pathways, all of which lead to the cleavage of C3 into biologically active C3a and C3b fragments [40]. Notably, an animal study has shown that C3 deficiency prevented the formation of choroidal neovascularization induced by the rupture of Bruch's membrane with laser photocoagulation in eyes of *C3* ^−/−^mice [48], indicating that C3 is a key factor in the development of choroidal neovascularization.

A nonsynonymous coding *C3* variant, rs2230199 (R102G), is strongly associated with AMD in populations of European descent, and this variant is presumed to be the most likely causal variant responsible for the disease association based on mechanistic functional evidence [11]–[13], [49], [50]. However, the association of R102G has not been reported in Asian populations [25]–[28], and allele frequencies of R102G vary widely among different ethnicities. For example, the risk allele is absent in Japanese [29] and rare (<1%) in Chinese populations [25], [27], [28], while the corresponding rate in Caucasians is 20% [25]. In the present study, we have found that a more common SNP of *C3*, rs2241394, is associated with AMD in a Japanese population. This association has not been documented by any previous genetic studies of AMD in European populations. These findings suggest that the susceptibility conferred by the R102G variant does not transcend ethnic lines and that there may be a significant difference in disease susceptibility loci in the *C3* region of populations of European and Asian descent. Notably, rs2241394 has previously been reported in a Japanese population to be associated with polypoidal choroidal vasculopathy [26], a major subphenotype of wet AMD in East Asian populations [51]–[53], and the direction of association was consistent with our findings. However, suggestive evidence for association of rs2250656 with wet AMD previously reported in a Chinese cohort [27] was not detected in the present study. We sought further evidence from a recent genome-wide association study of wet AMD in Japanese populations [29]; however, the arrays used in this study (Illumina HumanHap610-Quad BeadChip and Illumina HumanHap550v3 Beadchip) did not suit rs2241394.

The *C3* variant rs2241394 is an intronic SNP, and there is currently no evidence supporting its functional relevance. Using the FASTSNP program [37], we investigated potential functions of rs2241394. According to the analysis, this SNP was identified as lying in an intronic enhancer region created by a “C→G” change at rs2241394 that may lead to the creation of a binding site for the transcriptional factor *GATA-1*. Therefore, this SNP may have a functional relevance to disease risk for Japanese populations in the absence of surrounding 1000 Genome Project and HapMap SNPs that are highly correlated with rs2241394; however, fine-mapping and resequencing efforts are required to identify any potential as yet unidentified variants of more functional relevance.

In conclusion, we report a significant association between wet AMD and a common noncoding *C3* variant in a Japanese population. Our study provides evidence that *C3* is a common AMD-associated locus that transcends racial boundaries and provides an impetus for more detailed genetic characterization of the *C3* locus in Asian populations.
